# Supplementary material for: Inhibition of Granule Cell Dispersion and Seizure Development by Astrocyte Elevated Gene-1 in a Mouse Model of Temporal Lobe Epilepsy
Source: Biomolecules. 2024 Mar 20;14(3):380. doi: 10.3390/biom14030380 (PMC10968595; doi:10.3390/biom14030380)
Supplement: Supplementary file 1 [file biomolecules-14-00380-s001.zip › biomolecules-2906628-supplementary.pdf]

Supplementary Materials

Inhibition of Granule Cell Dispersion and Seizure Development by Astrocyte Elevated Gene-1 in a Mouse Model of Temporal Lobe Epilepsy

Supplementary Figures S1 to S8

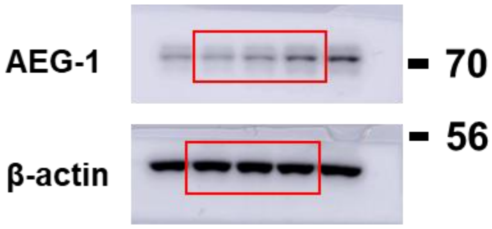

Figure S1. Full-length blots for Figure 1E

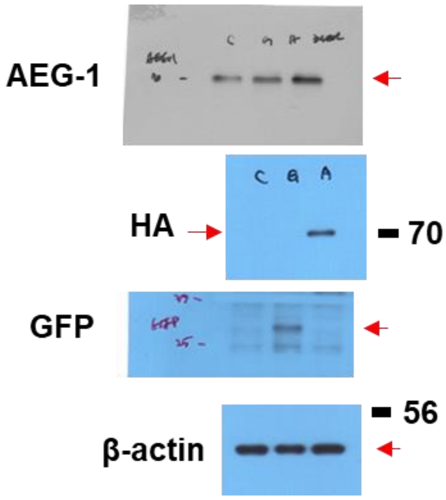

Figure S2. Full-length blots for Figure 2B

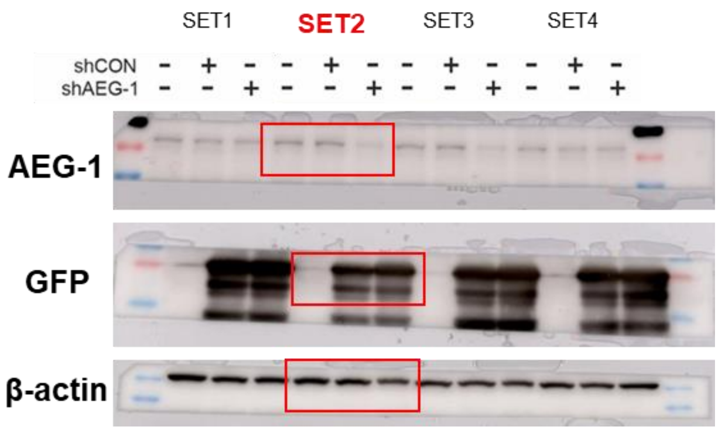

Figure S3. Full-length blots for Figure 2D

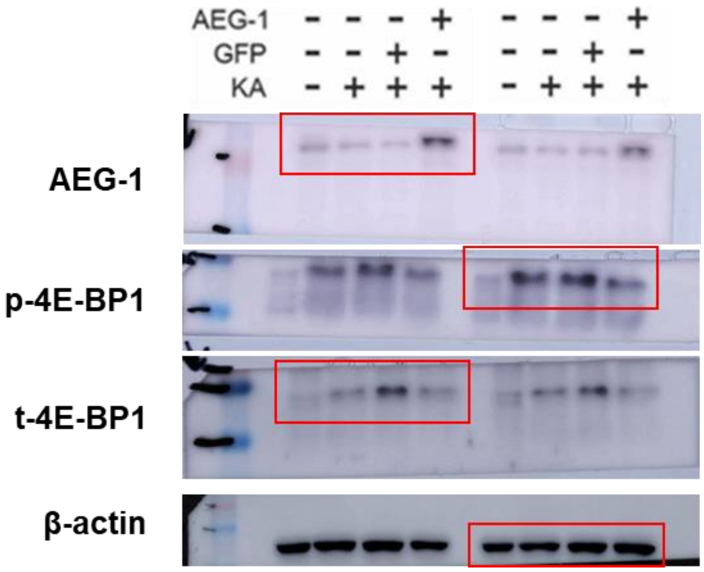

Figure S4. Full-length blots for Figure 3C

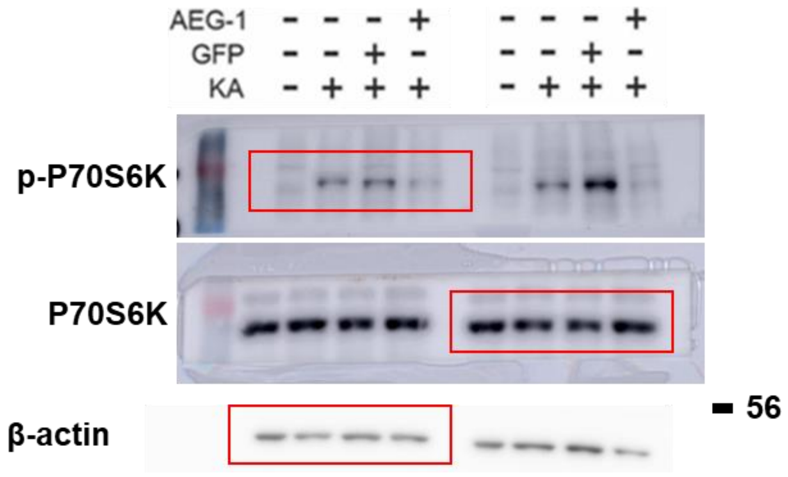

Figure S5. Full-length blots for Figure 3D

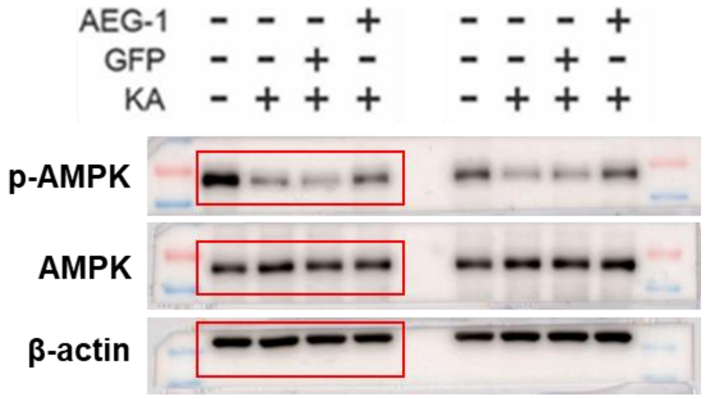

Figure S6. Full-length blots for Figure 3E

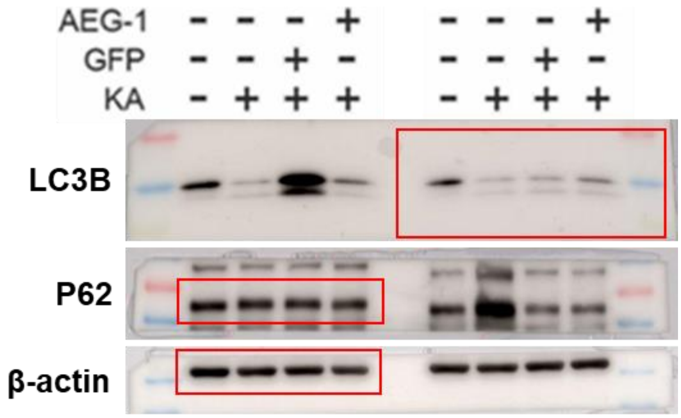

Figure S7. Full-length blots for Figure 3F

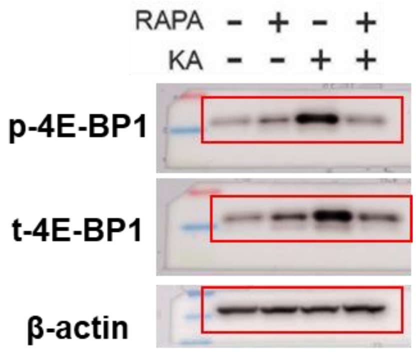

Figure S8. Full-length blots for Figure 4B
